# Case report: Post-thoracic surgery acquired venous thoracic outlet syndrome

**DOI:** 10.3389/fsurg.2023.1151921

**Published:** 2023-06-05

**Authors:** Mohamed Amine Mansouri, Jon Andri Lutz

**Affiliations:** Thoracic Surgery Unit, Department of Surgery, Hôpital Cantonal de Fribourg, Fribourg, Switzerland

**Keywords:** thoracic outlet syndrome, postoperative, thoracic surgery, etiology, management

## Abstract

Thoracic outlet syndrome (TOS) is a rare entity responsible for the vascular and/or nervous symptoms of the upper limbs. Unlike the congenital anatomical anomalies that cause TOS, acquired etiologies are even less common. Here, we report the case of a 41-year-old male with iatrogenic acquired TOS secondary to complex chest wall surgery for chondrosarcoma of the manubrium sterni; he was diagnosed with chondrosarcoma of the manubrium sterni in November, 2021. After staging was completed, primary surgery was performed. The operation was complex, with en-bloc resection of the manubrium sterni; the upper part of the corpus sterni; the first, second, and third bilateral parasternal ribs; and the medial clavicles, whose stumps were fixed on the first ribs. We reconstructed the defect using a double Prolene mesh, and bridged the second and third ribs on each side using two screwed plates. Finally, the wound was covered with pediculated musculocutaneous flaps. A few days after the operation, the patient presented with swelling in the left upper limb. Doppler ultrasound revealed slowing-down of the left subclavian vein flow, which was confirmed via thoracic computed tomography angiography. Systemic anticoagulation was initiated, and the patient began rehabilitation physiotherapy six weeks postoperatively. Symptoms had resolved by the 8-week outpatient follow-up, and anticoagulation therapy was stopped at three months; radiological follow-up demonstrated an improvement in subclavian vein flow without thrombosis. To the best of our knowledge, this is the first description of acquired venous TOS after thoracic surgery. Conservative treatment was found to sufficiently avoid the need for more invasive methods.

## 1. Introduction

Thoracic outlet syndrome (TOS) is caused by the compression of vascular and/or nervous elements bound for the upper limb at the superior thoracic aperture. It is a rare condition wherein the brachial plexus, subclavian artery, or vein is compressed at one or more of the three following anatomical locations of the thoracic outlet: the scalenic triangle; costoclavicular space; or, less often, the pectoralis minor space. Depending on the affected structure, TOS is specified as neurogenic (nTOS), arterial (aTOS), or venous (vTOS). The etiology is most often a congenital anatomical variation of the scalene muscles and/or costoclavicular osteoligamentary structures; however, acquired TOS can occur following trauma or intense solicitation of anatomical structures during physical sports or professional activities (1, 2).

Since TOS secondary to thoracic surgery is uncommon, we report a case of vTOS following a complex oncological thoracic wall surgery.

## 2. Case description

**Figure 1:**
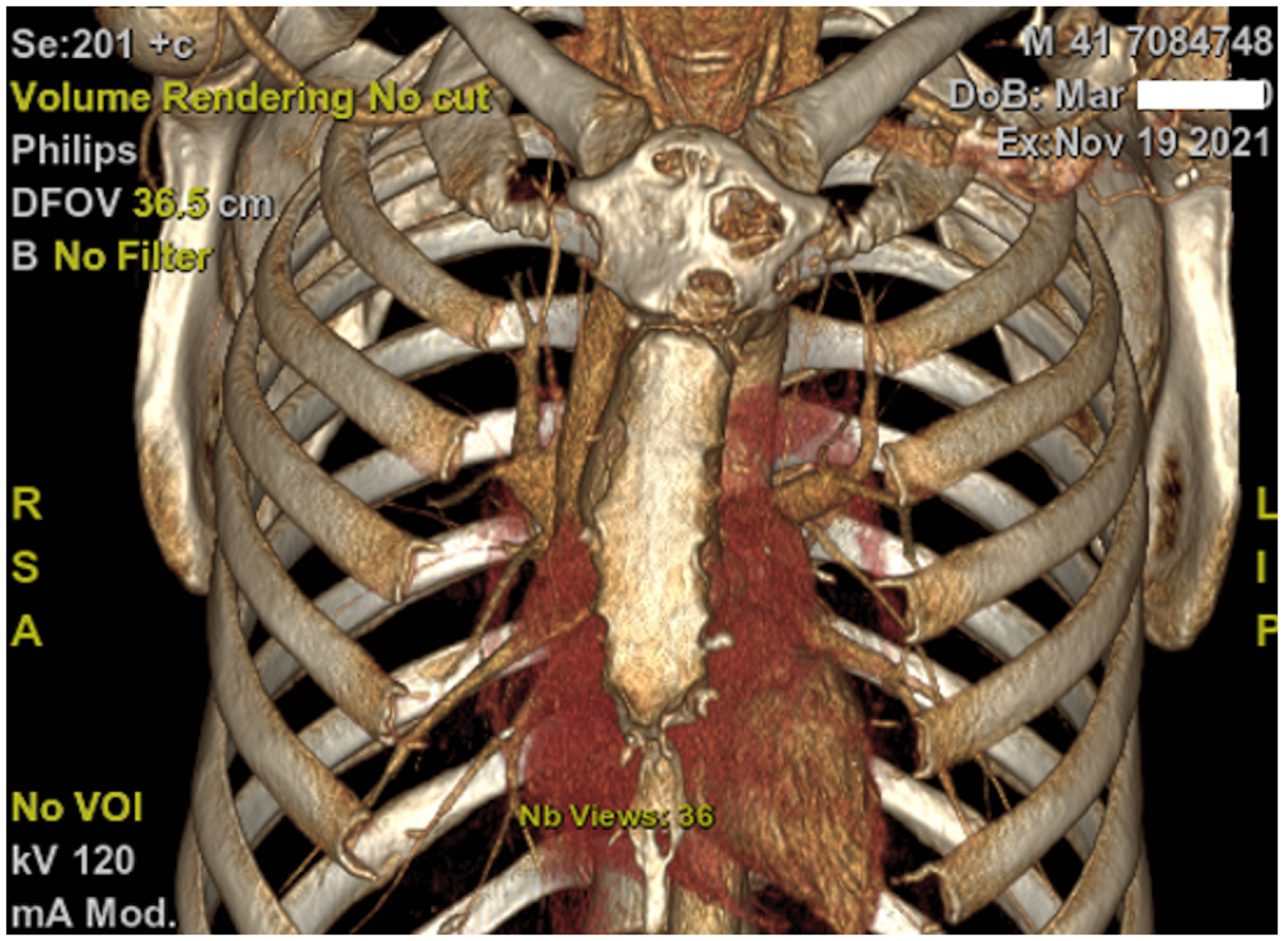
Computed tomography with three-dimensional reconstruction revealed a heterogeneous, cystic bony lesion limited to the manubrium sterni.

A 41-year-old male presented to our hospital in October 2021 with painful swelling near the sternoclavicular joint that had been evolving for two years. Despite conservative treatment prescribed by his family doctor, analgesic treatment, and physiotherapy, the suspected arthritis of the sternoclavicular joint did not improve. The pain mainly occurred due to physical activity in this patient, who worked as a mechanic on construction machinery. He did not report weight loss or any symptoms suggestive of a neoplastic disease. Physical examination revealed swelling on the left side of the manubriosternal angle without redness, and slight pain on deep palpation. Laboratory findings were normal. Thoracic computed tomography (CT) revealed a heterogeneous, cystic bony lesion limited to the manubrium sterni. The cortices were eroded, and the maximum diameter of the lesion was 3 cm (Figure 1). The diagnosis of chondrosarcoma of the manubrium sterni was confirmed using CT-guided biopsy. We completed the workup with a chest MRI that showed no infiltrative behavior or further manifestations of the tumor.

## 3. Diagnostic assessment

**Figure 2:**
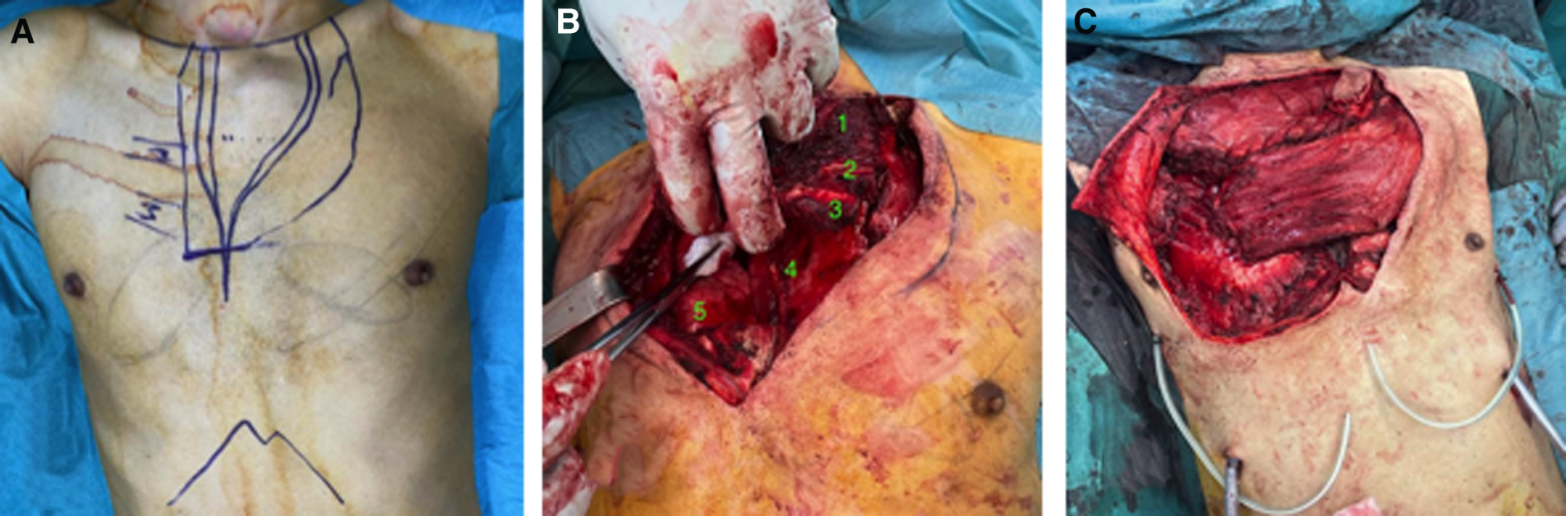
(**A**) Skin incision planning. (**B**) En-bloc resection of the manubrium sterni; upper part of the corpus sterni; first, second, and third bilateral parasternal ribs; and the medial clavicles, whose stumps were fixed on the first ribs. 1. Manubrium sterni tumor; 2. Second left rib; 3. Third left rib; 4. Mediastinal fat; 5. Right lung. (**C**) Musculocutaneous flaps covering chest wall reconstrauction.

We discussed the patient's case with the institutional board for sarcoma, and decided to perform primary resection and reconstruction. Complex oncological en-bloc resection and functional reconstruction of the anterior chest wall was performed by an interdisciplinary team of thoracic, orthopedic, and plastic surgeons on 7 February 2022. The manubrium sterni was removed together with the upper part of the corpus sterni; its cutaneous covering; the medial first, second, and third bilateral parasternal ribs; and the medial clavicles to achieve a sufficient resection margin (Figure 2). The left and right internal mammary arteries were then ligated. The reconstruction began with bilateral fixation of the clavicular stumps on the first ribs using long-chain, ultra-high molecular weight polyethylene tape (FiberWire®, Arthrex®, Florida, USA). We reconstructed the bony thoracic wall defect with a double Prolene mesh combined with two bridging universal 24-hole titanium plates (MatrixRIB™ Fixation System, De Puy Synthes, USA) on the second and third ribs. The anterior musculature of the neck was then inserted into the mesh, and the wound was finally covered with pediculated musculocutaneous flaps.

**Figure 3:**
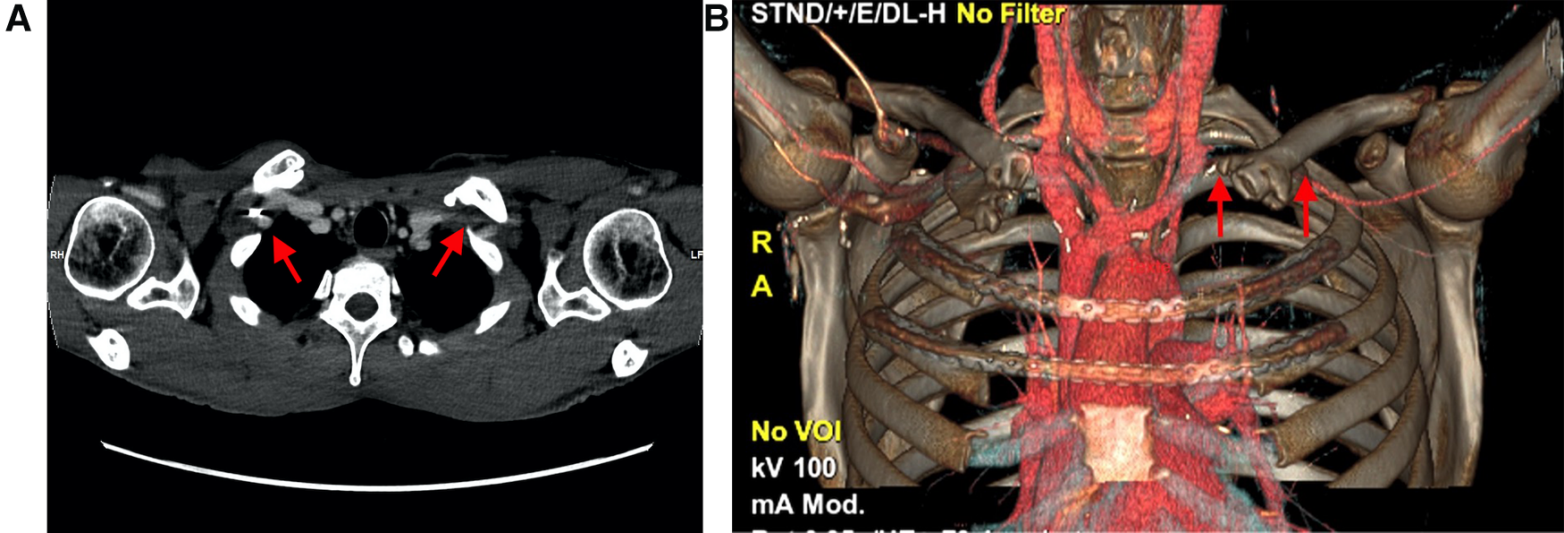
(**A**) Computed tomography revealed more narrowing at the level of the vein on the left side (5 mm) than the right side (11 mm). (**B**) Three-dimensional reconstruction indicating loss of contrast over the subclavian vein due to delayed opacification, secondary to stenosis.

Four days postsurgery, swelling and slight cyanosis of the left upper extremity appeared without numbness, decreased function of the arm or hand, or limb claudication or ischemia. Doppler ultrasound showed stenosis of the subclavian vein at the passage of the remaining clavicle, with a marked slowing-down of the flow in the arm veins, without thrombosis. The flow in the subclavian artery was normal with a triphasic profile. Thoracic CT angiography confirmed these findings, with more pronounced narrowing observed at the level of the vein on the left (5 mm) than right (11 mm) side. The space at the level of the artery was symmetrical, with a 10 mm distance on both sides (Figures 3A,B).

The patient was diagnosed with vTOS, and conservative treatment with systemic anticoagulation was initiated. The patient underwent rehabilitation physiotherapy in postoperative week six. The swelling of the left upper extremity gradually diminished, and all symptoms completely disappeared after 2 months. Anticoagulation was stopped three months postoperatively, with no recurrence of TOS to date. Follow-up Doppler ultrasound revealed a slight improvement in subclavian vein flow without thrombosis, while thoracic CT angiography revealed persistent left-sided costoclavicular pinching. At the one-year follow-up, the patient did not present with any symptoms of TOS.

## 4. Discussion

TOS is a rare clinical condition, with an incidence of 2–3 per 100,000 people per year (3). Neurological symptoms (nTOS) are the most frequent, appearing in approximately 90% of cases, followed by venous involvement (vTOS), and arterial involvement (aTOS), with a frequency of approximately 1% (4). The case presented above was exclusively vTOS, with symptoms of venous occlusion with no neurological or arterial involvement.

The scalenic triangle, which is lateral and slightly posterior, is where compressions of the subclavian artery and brachial plexus occur; the costoclavicular space, which is more anterior and medial, is where venous compressions occur (2). This is the most probable explanation for the postoperative vTOS that occurred in our patient. To achieve sufficient resection margins, we were required to shorten the left clavicle to a greater extent than the right due to the asymmetric location of the chondrosarcoma. Fixation of the clavicle stump on the first rib for reconstruction of the sternoclavicular joint reduced the costoclavicular space, thereby pinching the left subclavian vein.

The symptomatology in our patient was subacute venous compression syndrome without thrombosis, but with a marked slowing-down of the flow in the left upper extremity. Subclavian vein thrombosis would thus be expected, as it is the most common clinical presentation of vTOS. This occurs most often in young people who perform highly physical sports or professional activity soliciting the upper limbs, such as baseball players, or in individuals with a predisposed background of coagulopathy. This clinical presentation of exertional thrombosis of the subclavian vein is also known as Paget-Schroetter syndrome (5). Fortunately and likely due to early diagnosis and the rapid initiation of anticoagulation, as the patient was still in the ward, this complication could be avoided.

There are multiple etiologies for TOS; each is generally responsible for a single type of TOS. Some conditions, such as multiple exostoses, can result in a combination of nTOS and vTOS (6), while anatomical variations—such as the presence of a cervical rib or abnormalities in scalene muscles, fibroligamentous structure thickness, or insertion—may be responsible for TOS. Abnormal widening of the sternal (>20 mm) and vertebral (>15 mm) extremities of the first rib could also be a potential cause of TOS (7). Traumatic origins, such as fractures of the clavicle and/or the first rib, as well as cervical trauma (whiplash injury), are the most common causes of neurological symptoms in TOS. vTOS can appear after acute or chronic compression of the subclavian vein following intense strain on the upper limbs or compression of a hypertrophic bone callus from a fracture (1).To our knowledge, this is the first description of acquired vTOS secondary to complex chest wall surgery.

Treatment depends on the etiology and level of discomfort, and is generally conservative, especially for nTOS. Conservative measures include dedicated physiotherapy, weight control, education in correct posture, and pharmacological treatment, among others. In vTOS and aTOS, conservative measures should not delay necessary interventional or surgical treatment. Percutaneous mechanical thrombolysis can be useful in cases of vTOS with proven thrombosis (8), and while systemic anticoagulation is indicated to reduce the risk of thrombosis or recurrence, the optimal duration remains a topic of debate (5).

Given the absence of venous thrombosis in our case, conservative treatment with anticoagulation and physiotherapy was sufficient to reduce the symptoms, with no clinical or radiological recurrence observed at the one-year follow-up.

In conclusion, this is the first description of acquired venous TOS after thoracic surgery. Conservative treatment was found to sufficiently avoid the need for more invasive methods. Nevertheless particular attention should be paid to procedures that induce narrowing of the costo-clavicular space and the thoracic outlet in general. There is no much alternative for reconstruction of the costo-clavicular joint in this case, but early Doppler ultrasound could give a hint for starting early anticoagulation therapy.

## Data Availability

The raw data supporting the conclusions of this article will be made available by the authors, without undue reservation.
